# Tapentadol Prolonged Release for Severe Chronic Osteoarthritis Pain in the Elderly—A Subgroup Analysis of Routine Clinical Practice Data

**DOI:** 10.1155/2020/5759265

**Published:** 2020-04-14

**Authors:** Andreas Schwittay, Melanie Sohns, Birgit Heckes, Christian Elling

**Affiliations:** ^1^Practice for General Medicine, Special Pain Therapy & Palliative Medicine, Leipziger Str. 2, 04564 Böhlen, Germany; ^2^Grünenthal GmbH, Zieglerstr. 6, 52078 Aachen, Germany

## Abstract

**Background:**

Tapentadol prolonged release (PR) has been shown effective and generally well tolerated in a broad range of chronic pain conditions. This subgroup analysis investigated its benefits for elderly patients with severe chronic osteoarthritis (OA) pain in routine clinical practice. *Patients and Methods*. Data of all patients with chronic OA pain were extracted from the database of a prospective, 3-month noninterventional tapentadol PR trial. The data of elderly OA patients (>65 years of age; *n* = 752) were compared with the data of younger OA patients (≤65 years; *n* = 282).

**Results:**

Almost all patients (elderly 98.7% and younger patients 99.3%) had received long-term analgesic medication prior to the start of tapentadol PR treatment but presented with severe pain accompanied by considerable impairments in sleep quality and quality of life measures. Tapentadol PR provided effective pain relief in both patient groups, with slightly better outcomes in younger patients. However, the mean baseline pain intensity of 7.1 (SD 1.5) was reduced by 3.8 points (*p* ≤ 0.001), and sleep and quality of life measures had also markedly improved in the elderly: quality of sleep by 3 points, quality of life by 3.4 points, social activities by 3 points, and independence by 2.7 points (*p* ≤ 0.001 for all measures; 11-point scale). At the end of observation, 68% of the elderly had clinically relevant pain reductions of at least 50% (vs baseline), and 87.9% attained either their intended pain reduction target and/or an additional individual treatment target (both predefined during baseline examination). Only 8.4% of the elderly experienced adverse drug reactions, most frequently nausea (2.7% of patients) and dizziness (1.5%).

**Conclusion:**

Tapentadol PR provided effective and well-tolerated treatment of severe chronic OA pain for elderly patients in routine clinical practice. The favorable tolerability profile in particular suggests tapentadol PR as a treatment option before classical strong opioids are considered.

## 1. Introduction

Chronic pain is a common healthcare problem in the general adult population of the European Union and incurs high total population costs [1]. Many chronic pain patients are elderly, and this number is increasing in view of the aging population. Pain treatment is challenging in this age group; pain is often multifactorial and can be more difficult to assess, in particular, when communication and cognitive capacities decline [2]. Furthermore, multiple comorbidities can make the treatment of pain complex.

Tapentadol prolonged release (PR) has been shown to be effective and generally well tolerated in a broad range of chronic pain conditions [3]; trial data of up to 2 years of treatment also indicate good long-term safety and efficacy [4]. An analysis of routine clinical practice data since the introduction of tapentadol confirms the favorable overall safety profile of the medication [5]. In the management of moderate to severe chronic painful osteoarthritis (OA), one of the leading causes of disability in the elderly [6], tapentadol PR provided beneficial effects in several clinical trials [4, 7–13] including trials and subgroup analyses in the elderly [11–13]. The comparison to oxycodone controlled release (CR) suggested better pain relief and a more improved overall health status under tapentadol PR treatment [14]. The tolerability profile, in particular regarding gastrointestinal side effects, was also more favorable [12–14]. The latter is thought to be a consequence of a reduced *μ*-opioid load [15] due to the combination of two synergistic mechanisms of action (*μ*-opioid receptor agonism and noradrenaline reuptake inhibition) within the tapentadol molecule [16]. To investigate the administration of tapentadol PR in elderly patients under real-world clinical management conditions, we extracted the data of all patients with chronic OA pain from the database of a noninterventional tapentadol PR trial [17] and compared the data of elderly OA patients (>65 years of age) with the data of younger OA patients (≤65 years).

## 2. Patients and Methods

The presented data were extracted from the database of a prospective, noninterventional trial conducted between October 2010 and June 2011 in routine clinical practice (general practitioners and internists) in accordance with Section 4, Subsection 23, sentence 3 and Section 67, Subsection 6 of the Medicinal Products Act of the Federal Republic of Germany [17]. As required by law, the German Federal Institute for Drugs and Medical Devices, the German Association of Statutory Health Insurance Physicians, and the German Federal Associations of Health Insurance Funds were notified of the trial. The trial investigated the routine administration of tapentadol PR in clinical practice. Adult patients with severe chronic pain, who—in the opinion of the treating physician—were indicated for tapentadol PR treatment, could be enrolled. All treatment decisions were solely at the discretion of the treating physician. Tapentadol PR was prescribed in accordance with the summary of product characteristics current at the time [18] depending on previous treatment and pain severity. Adjustment of initial tapentadol PR doses within 3 days was recommended in case of insufficient analgesia (maximum recommended daily dose of 500 mg).

### 2.1. Data Collection

A total of 1432 medical centers in Germany were involved in data collection in the original trial [17]. Physicians documented data on case report forms during consultation at three time points: baseline examination, treatment follow-up after 4–6 weeks, and at end of observation after approx. 3 months. Documentation included patient demographics, pain diagnosis, previous long-term analgesic treatment, concomitant diseases, the reason for prescribing tapentadol PR, tapentadol dosages, and concomitant analgesics and co-analgesics and adjuvants. Patients were asked about their average pain intensity during the past 3 days, the course of pain over the previous 24 h, and impairments of sleep quality, quality of life, social activities, and independence in the previous 4 weeks. They rated these outcome measures on an 11-point numerical rating scale (NRS-11), with 0 = no pain to 10 = worst imaginable pain for pain intensity and 0 = no impairment to 10 = maximally conceivable impairment for pain-related impairments. During baseline examination, physician and patient jointly decided on treatment targets to be achieved during the treatment period. Apart from a pain intensity target (NRS-11), they specified an additional—realistically achievable—individual treatment target from the following areas: quality of life, physical functioning, mental well-being, and others. At the end of observation, this target was rated by the patients using a goal attainment scale (“much better than expected,” “better than expected,” “as expected,” “worse than expected,” or “much worse than expected” [19]). Successful tapentadol PR treatment was determined by (a) attainment of at least 50% reduction in pain intensity, (b) attainment of intended pain intensity, (c) attainment of additional individual treatment target, and (d) attainment of (b) and/or (c) (combined response).

Tolerability was assessed by monitoring the occurrence of adverse drug reactions (ADRs; possibly, probably, or definitely related to the trial medication) during the entire observation period (query during visits and spontaneous reports by the patients). These events were recorded on ADR documentation forms. Physicians did not make explicit causality evaluations. Completing the ADR documentation form implied that physicians assumed a causal relationship between the symptoms and tapentadol treatment; that is, they suspected an ADR.

At the end of treatment, physicians evaluated different aspects of tapentadol PR treatment (analgesia, tolerability, balance between efficacy and tolerability, compliance, quality of life, and overall treatment success), and both patients and physicians rated changes in the general condition of the patient since baseline examination.

### 2.2. Statistical Analysis

For the original trial, completed case report forms were collected by the sponsor from the participating physicians and forwarded to factum GmbH (Offenbach, Germany) for data processing (double data entry) using the data management program DMSys (version 5.1., SigmaSoft International, Los Gatos, CA, USA) and data check (for completeness, consistency, and plausibility).

All patients with painful OA of the knee and/or hip participating in the original trial were stratified into an elderly age group (>65 years of age) and a younger age group (≤65 years of age). Data extraction and effectiveness analysis were performed by factum GmbH (Offenbach Germany) using the statistics program SPSS (version 15.0.0; SPSS Inc., Chicago, USA).

Data were analyzed descriptively; percentages throughout the article are based on the total number of patients in the respective group (elderly, *n* = 752, or younger patients, *n* = 282) as denominator, unless otherwise stated. *p* values for changes in pain intensity, quality of sleep, quality of life, social activities, and independence over the course of the trial were calculated using Wilcoxon matched-pairs signed-rank test. Only patients with data at baseline and final visit were included in calculations for changes in these parameters and in calculations of treatment success criteria.

ADRs were encoded with the Medical Dictionary for Regulatory Activities (MedDRA) version 20.1.

## 3. Results

### 3.1. Patient Disposition and Demographics

In total, 1038 patients (33.1% of the original trial population [effectiveness set *n* = 3134]) experienced painful OA. Stratification by age resulted in an elderly group (>65 years of age) of 752 patients (72.4%) and a younger group (≤65 years of age) of 282 patients (27.2%); information regarding age was not available for 4 patients. Thus, 1034 patients were included in effectiveness and tolerability analyses.  Table 1 lists the baseline characteristics of both groups. The majority of the elderly were female; gender was more balanced in the younger patient group. More than 55% of patients in both groups had experienced pain for more than two years. Many patients with painful knee and/or hip OA also suffered from other pain conditions, most commonly low back pain (LBP; elderly 83.9%/younger group 83.3%). The majority (94.2%/80.1%) also had concomitant diseases; cardiovascular (79.3%/46.5%) and metabolic disorders (41.8%/39.4%) were most common. Hospitalization due to pain during the 12 months prior to tapentadol PR treatment was documented for 29.7% (223/750) of the elderly and 23.5% (66/281) of the younger group.

**Figure 1:**
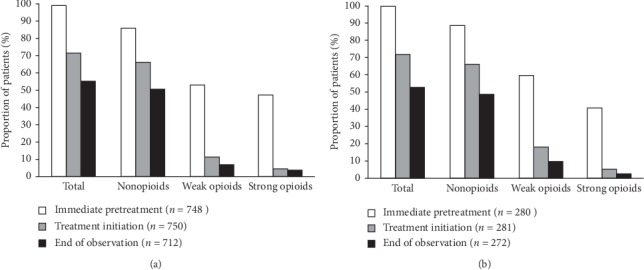
Long-term analgesics immediately preceding initiation of tapentadol PR treatment and change in concomitant analgesics over time. Multiple responses permitted. *n* number of patients with available data. (a) Osteoarthritis patients >65 years of age. (b) Osteoarthritis patients ≤65 years of age.

Nearly all patients (98.7% [738/748]/99.3% [278/280]) had already received long-term analgesic treatment prior to the start of tapentadol PR treatment (Figure 1), mainly consisting of several analgesics (Table 2). A higher proportion of elderly patients received transdermal fentanyl and buprenorphine compared with the younger group.  Table 3 lists the different dosages of strong opioids used immediately prior to tapentadol initiation. In addition, 34.2% (256/748) of the elderly and 27.1% (76/280) of the younger patients took analgesic rescue medication (Table 4). Table 4 also lists the proportion of patients with co-analgesics and adjuvant medications.

### 3.2. Treatment with Tapentadol PR

Main reasons why patients were switched to tapentadol PR were insufficient analgesia (elderly 90%/younger patients 89.7%) and insufficient quality of life (70.3%/75.4%; *n* = 751/*n* = 281; multiple responses permitted). Additional reasons were tolerability issues (39.4%/35.6%), insufficient balance between efficacy and tolerability (31.6%/30.3%), lack of compliance (11.9%/15%), and drug interactions with concomitant medication (7.3%/7.5%).

Mean daily tapentadol PR dosages were comparable between the groups at start of treatment but increased to a greater extent over the observation period in younger patients (Table 5). Three patients (two of them elderly) received higher than recommended daily doses of 500 mg (600 mg 2 patients and 750 mg one patient). The mean treatment duration was 87 ± 29.6 days for the elderly (*n* = 745) and 92.3 ± 28.9 days for younger patients (*n* = 272). The majority of patients in both groups continued tapentadol PR treatment after the treatment follow-up visit at 4–6 weeks (89.2% [667/748]/93.6% [262/280]) and after the 3-month visit (80.5% [603/749]/82.1% [230/280]). Main reasons for treatment discontinuation were unsatisfactory analgesia (8.4%/6.4%), the occurrence of ADRs (6.8%/4.3%), and no further requirement for analgesic treatment (e.g., surgery; 2.5%/5.7%; *n* = 751/*n* = 280). Nineteen elderly (2.5%) and 3 younger patients (1.1%) discontinued treatment due to hospitalization. Reasons included surgery (7 patients), fall (1), stroke (1), coronary heart disease (1), chronic fistula (1), ataxia (1), decompensated diabetes mellitus (1), exsiccosis/refusal to eat (1), and not specified (2). Pain was documented as the reason for hospitalization in four patients (all elderly; colitis was additionally listed for one of them); another elderly patient was hospitalized for periradicular therapy. Only 15 elderly patients (2%) and 2 younger patients (0.7%) discontinued within the first 10 days of tapentadol PR treatment. All cited either the occurrence of ADRs (1.9%/0.7%) and/or unsatisfactory analgesia (0.7%/0.4%) as reason for discontinuation. Initial daily dosing for these patients had been 2 × 50 mg (9 elderly, 2 younger patients), 2 × 100 mg (5 elderly), and 2 × 150 mg (1 elderly).

Given that tapentadol was only available for use to physicians for a limited time period prior to the start of the trial, the doses used in this trial were conservative. Nevertheless, the intake of additional long-term analgesic medication could be reduced over the observation period (Figure 1). At the start of tapentadol PR treatment, 71.2%/71.5% of patients received additional long-term analgesics (mainly nonopioids), and 20.3% in both groups received only tapentadol PR, and 8.5%/8.2% received tapentadol PR plus rescue medication (*n* = 750/*n* = 281). At the end of observation, the proportion of patients with tapentadol PR monotherapy had increased to 32.2% and 36.8%, respectively, and an additional 12.9%/10.7% received tapentadol PR as only long-term medication plus analgesic rescue medication (*n* = 712/*n* = 272). Additional long-term analgesics consisted mainly of nonopioids (Figure 1). Intake of rescue medication, antidepressants, antiepileptics, antiemetics, and laxatives had declined at the end of observation compared with data prior to tapentadol PR administration (Table 4).

### 3.3. Treatment Effects

**Figure 2:**
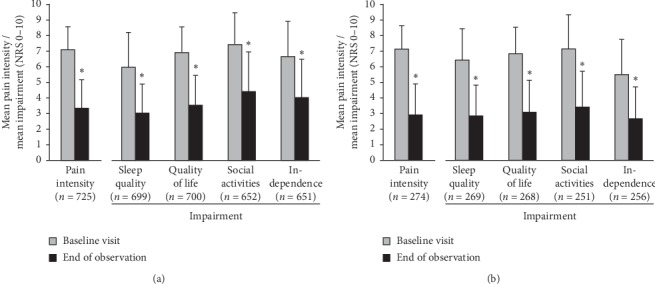
Changes in pain intensity and pain-related impairments of quality of sleep and quality of life parameters (+SD) over the observation period. Only patients with data for both observation time points were included. ^*∗*^*p* ≤ 0.001 compared with baseline visit (Wilcoxon matched-pairs signed-rank test). NRS, numerical rating scale. (a) Osteoarthritis patients >65 years of age. (b) Osteoarthritis patients ≤65 years of age.

During the 3-month observation period, mean baseline pain intensity of 7.1 ± 1.5 for both groups was reduced by 3.8 points in the elderly (*p* ≤ 0.001; Figure 2(a)) and by 4.2 points in the younger group (*p* ≤ 0.001; Figure 2(b)). Pain relief was also observed over the course of 24 h. Fewer elderly patients experienced constant pain with pain attacks (53.1% before tapentadol PR treatment and 12.5% at the end of observation), and pain-free intervals had markedly increased (from 6.1% to 51% of patients; ratings at both visits for 576 patients). The data for the younger age group showed a decrease in constant pain with pain attacks from 51.9% to 12.7%, and an increase in pain-free intervals from 8% to 60.9% (ratings at both visits for 212 patients).

At baseline examination, quality of sleep and all quality of life parameters were markedly impaired in both groups; social activities were affected most (Figure 2). All had considerably improved at the end of observation: quality of sleep by 3 points (elderly) and by 3.6 points (younger patients), quality of life by 3.4/3.7 points, social activities by 3.0/3.7 points, and independence by 2.7/2.8 points (all *p* ≤ 0.001; Figure 2). The proportion of patients with uninterrupted sleep increased from 5.9% to 26.6% in the elderly (ratings at both visits for 542 patients) and from 4.1% to 30.3% in the younger group (ratings at both visits for 218 patients).

### 3.4. Treatment Success

All four criteria for treatment success were attained by the majority of patients in both groups (Table 6). The mean targeted level of pain intensity at the end of the observation period as defined at baseline was 3.0 ± 1.2 by the elderly and 2.7 ± 1.5 by the younger group. This target was achieved or exceeded by 65.1% (472/725)/65% (178/274) of the patients. The most commonly chosen additional individual treatment targets were from the following areas: quality of life (73.3% [537/733]/73.1% [198/271]) and physical functioning (47.2% [346/733]/53.5% [145/271]; multiple responses possible). More than 85% of patients in both groups attained their chosen treatment target and the combined response target (Table 6).

### 3.5. Tolerability

Sixty-three elderly patients (8.4%) and 18 patients in the younger age group (6.4%) experienced ADRs during tapentadol PR treatment. The most frequently reported incidences were gastrointestinal and nervous system disorders (Table 7). “Drug withdrawal syndrome” or “withdrawal syndrome” was noted for 4 patients, two of them elderly. The three patients receiving daily tapentadol PR doses over 500 mg did not experience ADRs.

Serious ADRs were reported for 8 elderly patients (1.1%; 15 ADRs) and 2 younger patients (0.7%; 3 ADRs). In the elderly group, three patients had one ADR (fall, drug ineffective, and severe cardiac failure), four patients had two ADRs (severe diarrhea + severe sepsis, moderate abdominal pain upper + moderate nausea, ataxia + withdrawal syndrome, and moderate abdominal pain + colitis), and one patient had four moderate ADRs (abnormal sensation in eye, eye movement disorder, vision blurred, and visual impairment). In the younger age group, drug withdrawal syndrome was documented for one patient and severe fecaloma + severe ileus paralytic for a second patient.

### 3.6. Evaluation of Treatment

At the end of observation, tapentadol PR treatment was rated “good” or “very good” for the majority of patients (Table 8). Physicians found the general condition of their patients much or very much improved in 75.2% [533/709] of elderly patients and in 79.7% [212/266] of younger patients. Patients' ratings were slightly less positive (69.1% [476/689] elderly and 75.9% [195/257] younger group).

## 4. Discussion

The elderly OA patients analyzed here not only presented with painful knee and/or hip OA but also were afflicted by other pain conditions, most notably LBP. Many had been suffering from pain for more than 2 years, and almost all had received long-term analgesic medication before the start of tapentadol PR treatment. Despite this use of, often strong, analgesics (47% of patients were administered WHO III medication), the burden of pain was high: mean pain intensity at baseline was rated as severe and accompanied by considerable impairments in sleep quality and daily functioning, and thus markedly reduced quality of life. Not surprisingly, the main reasons physicians opted for a switch to tapentadol PR were insufficient analgesia and quality of life with previous analgesic treatment.

The 3-month treatment with tapentadol PR provided effective pain relief in both patient groups with slightly better outcomes in younger patients. However, a total of 68% of the elderly patients had clinically relevant pain reductions of at least 50% (vs baseline), and 65% achieved their intended pain intensity target. Mean pain intensity at the end of observation deviated by only 0.4 points from the mean treatment target of 3.0 points defined at baseline visit. The results are comparable to findings for the original trial population (72%/66% [17]) and the large population of a second 3-month noninterventional tapentadol PR trial (65%/60% [20]).

Nausea, dizziness, and constipation were most frequently reported by the 8.4% of elderly patients experiencing ADRs during tapentadol PR treatment. Only 6.8% discontinued treatment due to the occurrence of an ADR. Physicians considered tapentadol PR treatment well tolerated by the majority of elderly patients, with “good” to “very good” ratings of over 80% in the following categories: gastrointestinal tolerability, CNS tolerability, and general tolerability.

Patients suffering from severe pain frequently complain about impairments in daily life such as diminished sleep quality, restrictions in social activities, and loss of independence. These impairments may lead to social isolation, anxiety, and depression, and may thus greatly affect their quality of life. Results for Germany from the 2010 National Health and Wellness Survey showed that the impact of severe and frequent pain on the quality of life is greater than the contribution of sociodemographic factors, the presence of comorbidities, or health risk factors such as obesity, alcohol, and smoking [21]. OA pain limits functionality. Physical activity is often restricted, which is considered a major factor in the onset of frailty in the elderly [22], resulting in a loss of independence and ultimately a move to a care facility. Thus, more so than in any other age group, pain relief in the elderly may, for some patients, be key to a longer independent and involved life. In addition, it is also likely to have an impact on the burden of care for their families and costs arising for society in general. These impairments of functionality were observed in our elderly OA patients at baseline. They presented with considerable impairments in sleep quality and quality of life at baseline, with social activities being the most severely impacted quality of life measure. Not surprisingly, the majority considered improvements in quality of life an important treatment target: 73% of the patients chose an additional individual treatment target from this domain. Effective pain relief under tapentadol PR treatment was accompanied by marked improvements in all quality of life measures which contributed to the overall treatment success of the medication. It should also be noted that one-third of the elderly patients (32.2%) did not require additional long-term analgesic medication at the end of observation and a further 12.9% received only tapentadol PR plus analgesic rescue medication.

Elderly OA patients received mean daily tapentadol PR doses of 199 ± 101 mg, on average 14 mg less than the younger age group (213 ± 103 mg) at the end of observation and less than half the maximum recommended daily dose of 500 mg. This dosage was at the lower end of the mean daily dose range observed for other routine clinical practice tapentadol PR treatment data in cancer and noncancer pain (192–287 mg [17, 20, 23–27]). In contrast, mean daily doses in phase 3 clinical trials were approx. 300 mg [3] and thus above the higher range found in noninterventional trials. Most of the clinical practice trials had been initiated when tapentadol PR was newly authorized and physicians had not yet gained experience with dosing this new analgesic. They might thus have relied more on additional analgesics instead of using the full tapentadol PR dosing range. Adherence to dose adjustment recommendations available today might further improve therapeutic outcomes including a reduction in the requirement for additional long-term analgesics, an advantage for patients with polypharmacy.

Overall, tapentadol PR was effective and well tolerated in the treatment of painful OA in the elderly in routine clinical practice. The findings complement the positive results of tapentadol PR treatment in elderly patients ≥65 years and ≥75 years of age obtained in pooled analyses of randomized controlled clinical trials [12, 13].

Analyses of routine clinical practice data are limited by the absence of a control group. However, our data which derived from a prospective, noninterventional trial where treatment decisions were solely at the discretion of the treating physician provide an insight into real-world pain management and complement the findings of randomized controlled trials with predefined trial criteria. For example, in contrast to randomized controlled trials with balanced group sizes, the proportion of elderly patients in our analysis was much higher (reflecting the real-life situation). However, this did not present a problem as no direct statistical comparison between groups was presented and the large number of patients in both groups allowed for a meaningful analysis within both. The lack in balance of potential confounding factors such as gender (higher proportion of women in elderly compared with younger age group) represents a limitation of our analysis, as this makes the groups not directly comparable to each other and might be reflected in the results of the two groups. However, the focus of our analysis was on the effectiveness and safety of tapentadol in elderly patients in a real-life situation in contrast to what is observed in younger patients in real-life rather than a direct comparison of tapentadol PR efficacy between both groups. It should be noted that most patients in this analysis not only suffered from painful OA but also from LBP. Data analysis could not distinguish between the two pathologies. The coexistence of these two pathologies is often observed in clinical practice and is not surprising, given that one could be caused by the other, for example, insufficient pain treatment of OA might lead to issues with posture resulting in subsequent complaints in other body regions such as the lower back.

## 5. Conclusion

Tapentadol PR provided effective and well-tolerated treatment of severe chronic OA pain for elderly patients in routine clinical practice. The marked improvements in all quality of life measures and the favorable tolerability profile in particular suggest tapentadol PR as an early treatment option before classical strong opioids are considered.

## Figures and Tables

**Table 1:** Baseline characteristics of the trial populations.

	Patients aged >65 years		Patients aged ≤65 years	
	*n*		*n*	
Gender	752		282	
Male		206 (27.4%)		130 (46.1%)
Female		546 (72.6%)		152 (53.9%)
Age (years)	752	77.7 ± 6.5	282	56.2 ± 6.7
Body mass index (kg/m^2^)	744	29.5 ± 5.3	280	30.5 ± 6.7
Type of osteoarthritis	752		282	
Knee^a^		577 (76.7%)		204 (72.3%)
Hip^b^		476 (63.3%)		149 (52.8%)
Knee and hip		301 (40%)		71 (25.2%)
Duration of pain	751		282	
<1 month		8 (1.1%)		0
1–3 months		33 (4.4%)		16 (5.7%)
>3–6 months		52 (6.9%)		31 (11%)
>0.5–1 year		95 (12.7%)		32 (11.4%)
>1–2 years		112 (14.9%)		46 (16.3%)
>2 years		451 (60.1%)		157 (55.7%)
Type of pain	728		267	
Pure nociceptive pain		54 (7.4%)		15 (5.6%)
Pure neuropathic pain		13 (1.8%)		3 (1.1%)
Mixed pain		661 (90.8%)		249 (93.3%)

Data are mean ± SD or number of patients (%); ^a^all patients with knee osteoarthritis and knee/hip osteoarthritis; ^b^all patients with hip osteoarthritis and hip/knee osteoarthritis.

**Table 2:** Analgesic medication immediately prior to treatment with tapentadol PR.

	Aged >65 years (*n* = 752)	Aged ≤65 years (*n* = 282)
Strong opioids^a^	353 (46.9%)	113 (40.1%)
Fentanyl (transdermal)	129 (17.2%)	36 (12.8%)
Oxycodone/naloxone	74 (9.8%)	30 (10.6%)
Oxycodone	61 (8.1%)	26 (9.2%)
Buprenorphine	56 (7.5%)	9 (3.2%)
Morphine	37 (4.9%)	19 (6.7%)
Hydromorphone	33 (4.4%)	9 (3.2%)
Other	1 (0.1%)	1 (0.4%)
Weak opioids^a^	396 (52.7%)	166 (58.9%)
Tramadol	214 (28.5%)	100 (35.5%)
Tilidine/naloxone	200 (26.6%)	76 (27%)
Other/not specified	1 (0.1%)	0
Nonopioids^a^	641 (85.2%)	247 (87.6%)
Nonsteroidal anti-inflammatory drugs	468 (62.2%)	199 (70.6%)
Metamizole	330 (43.9%)	105 (37.2%)
Muscle relaxants	115 (15.3%)	64 (22.7%)
Paracetamol	62 (8.2%)	34 (12.1%)
Other/not specified	34 (4.5%)	9 (3.2%)

Data are number of patients (%); ^a^multiple responses permitted. PR, prolonged release.

**Table 3:** Dosages of strong opioids received immediately prior to treatment with tapentadol PR.

	Aged >65 years	Aged ≤65 years
Fentanyl (transdermal)	129	36
≤25 *μ*g/h	53	17
50 *μ*g/h	36	7
≥75 *μ*g/h	40	12
Oxycodone/naloxone	74^a^	30
≤40 mg/day	66	23
41–80 mg/day	7	6
>80 mg/day	0	1
Oxycodone	61^a^	26
≤40 mg/day	49	18
41–80 mg/day	8	7
>80 mg/day	3	1
Buprenorphine	56^a^	9
≤20 *μ*g/h	33	9
35 *μ*g/h	12	0
52.5 *μ*g/h	6	0
70 *μ*g/h	4	0
Morphine	37	19^a^
≤60 mg/day	26	10
61–120 mg/day	8	7
>120 mg/day	3	1
Hydromorphone	33	9^a^
≤8 mg/day	8	4
9–16 mg/day	17	2
>16 mg/day	8	2

Data are number of patients. ^a^Data missing for one patient. PR, prolonged release.

**Table 4:** Administration of co-analgesics and adjuvants.

	Immediately prior to tapentadol treatment	At the start of tapentadol treatment	At the end of observation
Patients aged >65 years (*n* = 752)			
Co-analgesics	299 (39.8%)	275 (36.6%)	249 (33.1%)
Antidepressants	229 (30.5%)	219 (29.1%)	205 (27.3%)
Antiepileptics	121 (16.1%)	97 (12.9%)	79 (10.5%)
No data	0	0	30 (4%)
Adjuvants	221 (29.4%)	197 (26.2%)	152 (20.2%)
Antiemetics	83 (11%)	70 (9.3%)	49 (6.5%)
Laxatives	170 (22.6%)	144 (19.2%)	120 (16%)
Adjuvants NOS	1 (0.1%)	2 (0.3%)	0
No data	3 (0.4%)	3 (0.4%)	38 (5.1%)
Rescue medication	256 (34%)	235 (31.3%)	212 (28.2%)
No data	4 (0.5%)	2 (0.3%)	39 (5.2%)
Patients aged ≤65 years (*n* = 282)			
Co-analgesics	133 (47.2%)	110 (39%)	92 (32.6%)
Antidepressants	111 (39.4%)	99 (35.1%)	81 (28.7%)
Antiepileptics	54 (19.2%)	36 (12.8%)	30 (10.6%)
No data	0	0	8 (2.8%)
Adjuvants	67 (23.8%)	53 (18.8%)	28 (9.9%)
Antiemetics	36 (12.8%)	28 (9.9%)	15 (5.3%)
Laxatives	45 (16%)	33 (11.7%)	20 (7.1%)
Adjuvants NOS	1 (0.4%)	1 (0.4%)	0
No data	1 (0.4%)	2 (0.7%)	13 (4.6%)
Rescue medication	76 (27%)	74 (26.2%)	70 (24.8%)
No data	2 (0.6%)	1 (0.4%)	10 (3.6%)

NOS, not otherwise specified. Data are number of patients (%).

**Table 5:** Daily tapentadol PR doses over the observation period.

	Aged >65 years (*n* = 752)	Aged ≤65 years (*n* = 282)
At the start of treatment (mg)	129.8 ± 62.0	132.5 ± 64.7
2 × 50 mg	564 (75%)	204 (72.3%)
2 × 100 mg	146 (19.4%)	57 (20.2%)
≥2 × 150 mg	33 (4.4%)	16 (5.7%)
Other	9 (1.2%)	5 (1.8%)
At the end of titration (mg)	181.5 ± 92.5	195.1 ± 93.3
No data	12 (1.6%)	4 (1.4%)
At the end of observation (mg)	198.6 ± 100.7	213.4 ± 103.4
No data	16 (2.1%)	5 (1.8%)

Data are mean ± SD or number of patients (%). PR, prolonged release.

**Table 6:** Proportion of patients who had attained one or several treatment targets at the end of the 3-month tapentadol PR treatment.

	Patients aged >65 years	Patients aged ≤65 years
(a) Attainment of at least 50% reduction in pain intensity vs. baseline	67.6% (*n* = 725)	74.1% (*n* = 274)
(b) Attainment of intended pain intensity	65.1% (*n* = 725)	65% (*n* = 274)
(c) Attainment of additional individual treatment target	87.8% (*n* = 713)	91.5% (*n* = 272)
(d) Attainment of (b) and/or (c) (combined response rate)	87.9% (*n* = 728)	92% (*n* = 274)

*n*, number of patients with valid data; PR, prolonged release.

**Table 7:** Most frequently reported adverse drug reactions (incidences of >1% of patients for each preferred term).

	Patients aged >65 years (*n* = 752)	Patients aged ≤65 years (*n* = 282)
Any adverse drug reaction	63 (8.4%)	18 (6.4%)
Gastrointestinal disorders	33 (4.4%)	12 (4.3%)
Nausea	20 (2.7%)	8 (2.8%)
Constipation	8 (1.1%)	1 (0.4%)
Nervous system disorders	24 (3.2%)	8 (2.8%)
Dizziness	11 (1.5%)	6 (2.1%)

Data are number of patients (%).

**Table 8:** Evaluation of tapentadol PR treatment by the treating physicians at the end of observation (proportion of patients with “good” or “very good” ratings).

	Patients aged >65 years (*n* = 752)	Patients aged ≤65 years (*n* = 282)
Analgesia	76.4% (*n* = 698)	85.2% (*n* = 270)
General tolerability	84.9% (*n* = 697)	89.7% (*n* = 272)
Gastrointestinal tolerability	85.9% (*n* = 695)	88.5% (*n* = 269)
CNS tolerability	86.5% (*n* = 691)	91.5% (*n* = 271)
Balance between efficacy and tolerability	82.5% (*n* = 690)	89.5% (*n* = 267)
Quality of life	70.8% (*n* = 694)	81.7% (*n* = 268)
Compliance	86.9% (*n* = 694)	89.5% (*n* = 266)
Overall treatment success	78.9% (*n* = 693)	85.6% (*n* = 270)

*n* = number of patients with valid data. CNS, central nervous system. PR, prolonged release.

## Data Availability

On a case-by-case basis, the authors will share on request tables of the subgroup analysis.

## Abbreviations

ADR: Adverse drug reaction
CR: Controlled release
LBP: Low back pain
NRS: Numerical rating scale
OA: Osteoarthritis
PR: Prolonged release
WHO: World Health Organization.
